# Bayesian-Driven First-Principles Calculations for Accelerating Exploration of Fast Ion Conductors for Rechargeable Battery Application

**DOI:** 10.1038/s41598-018-23852-y

**Published:** 2018-04-11

**Authors:** Randy Jalem, Kenta Kanamori, Ichiro Takeuchi, Masanobu Nakayama, Hisatsugu Yamasaki, Toshiya Saito

**Affiliations:** 1 Japan Science and Technology Agency (JST), PRESTO, 4-1-8 Honcho Kawaguchi, Saitama, 332-0012 Japan; 2 National Institute for Materials Science – Global Research Center for Environment and Energy based on Nanomaterials Science (NIMS-GREEN), 1-1 Namiki, Tsukuba, 305-0044 Ibaraki Japan; 3 Center for Materials research by Information Integration (CMI2), Research and Services Division of Materials Data and Integrated System (MaDIS), NIMS, 1-2-1 Sengen, Tsukuba, 305-0047 Ibaraki Japan; 4 Department of Computer Science/Research Institute for Information Science, Nagoya Institute of Technology, Gokiso-cho, Showa-ku, Nagoya, Aichi 466-8555 Japan; 5 RIKEN Center for Advanced Intelligence Project, 1-4-1 Nihonbashi, Chuo-ku, Tokyo, 103-0027 Japan; 6 Frontier Research Institute for Materials Science (FRIMS) & Department of Advanced Ceramics, Nagoya Institute of Technology, Gokiso-cho, Showa-ku, Nagoya, 466-8555 Aichi Japan; 7 Elements Strategy Initiative for Catalysts and Batteries, Kyoto University, f1-30 Goryo-Ohara, Nishikyo-ku, 615-8245 Kyoto Japan; 8 Battery Material Engineering & Research Div., Toyota Motor Corporation, 1200, Mishuku, Susono, 410-1193 Shizuoka Japan

## Abstract

Safe and robust batteries are urgently requested today for power sources of electric vehicles. Thus, a growing interest has been noted for fabricating those with solid electrolytes. Materials search by density functional theory (DFT) methods offers great promise for finding new solid electrolytes but the evaluation is known to be computationally expensive, particularly on ion migration property. In this work, we proposed a Bayesian-optimization-driven DFT-based approach to efficiently screen for compounds with low ion migration energies (${{\boldsymbol{E}}}_{{\boldsymbol{b}}}{\boldsymbol{)}}$. We demonstrated this on 318 tavorite-type Li- and Na-containing compounds. We found that the scheme only requires ~30% of the total DFT-${{\boldsymbol{E}}}_{{\boldsymbol{b}}}$ evaluations on the average to recover the optimal compound ~90% of the time. Its recovery performance for desired compounds in the tavorite search space is ~2× more than random search (i.e., for ${{\boldsymbol{E}}}_{{\boldsymbol{b}}}$ < 0.3 eV). Our approach offers a promising way for addressing computational bottlenecks in large-scale material screening for fast ionic conductors.

## Introduction

There has been a rapidly growing interest to systematically search for new fast ionic conductors using high-throughput calculations, particularly by leveraging from the wealth of material information from crystal structure databases^1–5^. The workhorse simulation tool that is often employed in these tasks has been based on first-principles density functional theory (DFT)^6,7^ which can offer a level of predictive accuracy that is comparable to experimental results^8–10^. However, DFT-based material search with transition state property criterion (e.g., ${E}_{b}$) is still few and of limited scope^11–14^. The relatively high calculation costs involved make these efforts very tedious and in most cases impractical^15–17^. Other cheaper methods have been utilized as substitutes and for rough screening, one of these is by force-field (FF) approach such as bond valence summation^18^. However, the drawback of FF is that its accuracy is strongly tied to the quality of its fitted empirical parameters and the choice of the functional forms used to approximate interatomic bonding potentials. The task of fitting for FF parameters, which relies on experimental and/or DFT data, is also time-consuming. As a result, it is technically challenging to obtain a truly robust FF parameter set that can be applied for a large variety of structures and chemistries.

The difficulty of keeping the computational cost manageable in DFT-based material search/screening has also made it prohibitive to readily expand the search space by ionic substitution, for example, in known database compounds. Nonetheless, there is a huge merit for checking these gaps in the composition space because they could be fertile grounds for new materials^19^. In fact, a number of discoveries up to date were realized even with only a select few number of substitutions, such as in the case of Li/Na ionic conductors: layered-type AMO_2_ (A: Li, M:Co, Ni,)^20,21^, olivine-type AMPO_4_ (A: Li; M: Fe, Mn)^22^, garnet-type Li_7_La_3_Zr_2_O_12_^23^, and tetragonal A_10_MP_2_S_12_ (A: Li, Na; M: Ge, Sn)^24,25^.

In order to take advantage of the accuracy of DFT for predicting transition state properties and to extend today’s material search for fast ionic conductors beyond the known database composition space, two major computational cost issues need to be tackled: (i) the inherent cost for calculating transition state properties itself (such as for ${E}_{b}$) and (ii) the cost due to the combinatorial complexity arising from ionic substitution in known structure types. Meanwhile, traditional regression/classification techniques are limited with issues in terms of material discovery: (i) fitting precision and uncertainty issue which is linked to the need for larger and larger training dataset as the search space also becomes larger (i.e., to improve out-of-sample prediction) and (ii) the cost of building sufficient training data especially for calculation-intensive target properties (eg., ${E}_{b}$, ionic conductivity in rechargeable Li ion batteries). Our proposed solution, as will be further explained later, is to formulate an appropriate search/screening strategy in which instead of exhaustive or random searches, calculation resources are selectively allocated on compounds that would likely demonstrate fast ionic conduction, or in the case here, compounds with low ${E}_{b}$ values. On the other hand, calculations for compounds with high ${E}_{b}$ values are to be minimized, if not totally avoided. This strategy can be formalized as the process of solving an optimization problem, but its objective function (i.e., for ${E}_{b}$) cannot be directly expressed analytically. Conventional optimization approaches such as convex optimization and gradient descent are not straightforward to implement in such cases.

Recently, machine learning algorithms based on Bayesian optimization (BO) have become increasingly popular for efficiently solving material science problems. Unique from traditional machine learning methods (eg., LASSO and neural network), BO constructs a probabilistic model for the objective function and then exploits this model for deciding the next query point to be evaluated. BO has been successfully used in crystalline interface optimization^26^, construction of interatomic potentials^27^, and low-energy region identification in a potential energy surface^28^. Studies have also shown to implement BO together with DFT in order to find single- and binary-component solids with high melting temperature^29^, compounds with low lattice thermal conductivity^30^, and ternary compounds with desired elastic properties^31^. These demonstrations are indeed a step towards a sound and efficient design of new materials.

Our present work is also aimed towards finding new materials in an enhanced iterative-driven manner, but this time the chemical search space is a quinary system with battery as the target application and with the use of a transition state property as a practical search criterion – ${E}_{b}$. Quinary system is a challenging but highly relevant search space for battery research because many relevant materials and their optimization lies in this composition space. Examples include Li_7−x_La_3_Zr_2−x_Ta_x_O_12_ solid electrolytes which has an optimized ionic conductivity on the order of 10^−3^ S/cm^32^, LiNi_x_Mn_y_Co_z_O_2_ cathodes which show good specific energy and specific power density^33^, and Na_3_Ti_2_P_2_O_10_F which is a new candidate anode material for sodium ion batteries^16,34,35^. Moreover, the ${E}_{b}$ criterion, which can also be experimentally accessed (eg., by impedance measurements and NMR), is a very important metric for battery researchers since it is ubiquitous in all of the critical device components (anode, cathode, and electrolyte). Previous efforts have dealt mainly on unary and binary systems, whereas the present study emphasized on formulating an efficient and automation-compatible property-based search/screening in the extended composition space of five-component compounds with a fixed crystal structure (tavorite AMXO_4_Z system, where A, M, X, and Z are sites for ionic substitution), covering yet-to-be synthesized chemistries that are not yet found in databases. The choice of tavorite AMXO_4_Z is motivated by the idea that it is relatively unexplored in terms of varying its composition, so there is a good possibility of finding truly unreported new compounds^36–38^. Another reason is that one of the reported compound, LiFeSO_4_F, demonstrates high Li insertion rate which means that pathways within the structure can be highly favorable for ion transport^36^. Ion migration property in crystalline solids (i.e., ${E}_{b}$), to the best of our knowledge, still has no published databases up to now (experimental or computational) and also, by DFT, incurs significantly higher calculation costs than, for example, thermodynamic property-based search criteria (as in some of the previous works mentioned above^26,28,29,31^). We also demonstrate concretely in this work the ability of BO for knowledge transfer in a successive screening scenario, that is, using the posterior from one screening task as a prior for the next screening task. Finally, we also aimed to devise a practical workflow for automated material search/screening that is flexible enough to handle a large number and variety of material descriptors, this is realized by coupling the workflow with a modified BO scheme that is general for high dimensions^39–42^. We then use the BO probabilistic model to find compositions of low ${E}_{b}$ for Li and Na ions within the database-reported ordered tavorite structure. The target application for the tavorite-type ionic conductors is for solid electrolyte use, so only compounds that do not permit electronic conduction are considered (i.e., no transition metals are included for ionic substitution).

## Results

### Chemical search space and crystal structure description

**Figure 1:**
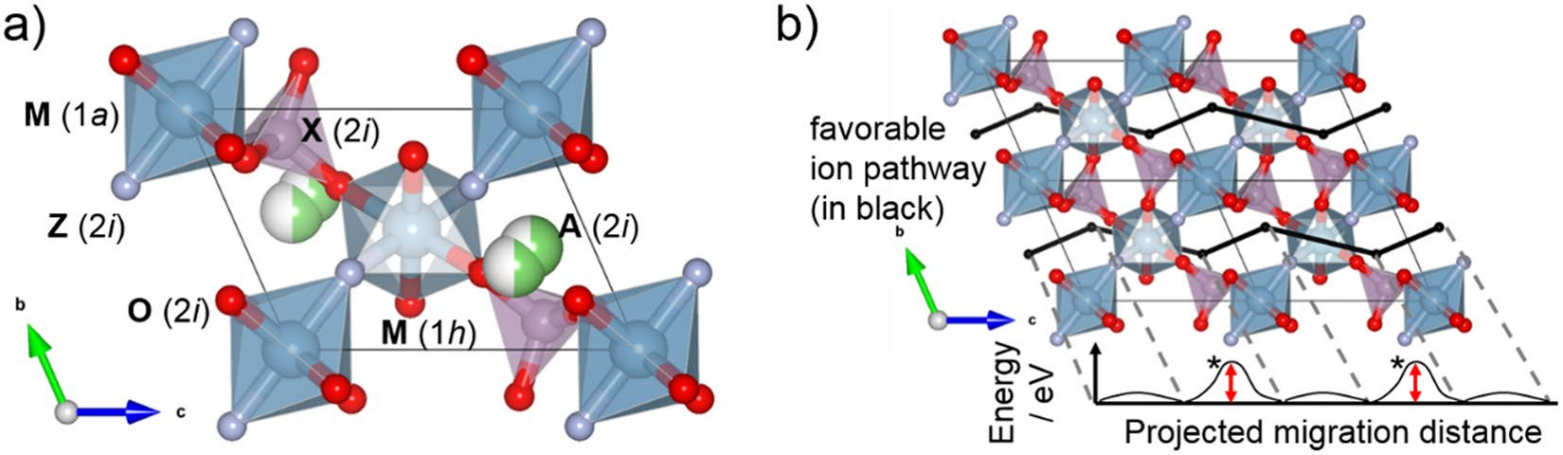
(**a**) Model unit cell for the tavorite AMXO_4_Z (*P* $\bar{1}$) showing various crystallographic sites and polyhedral units. Green/white spheres for A atoms indicate a splitting site. (**b**) The predicted favorable conduction pass for A cations within the tavorite framework (A atoms removed) as shown in its 1 × 2 × 2 supercell (in black, along *c*-direction) (13). The local barrier height ${E}_{b}$ marked by asterisks are equivalent characteristic path bottlenecks. The VESTA software was uses for structure visualization^43^.

Tavorite-type compounds with a general formula AMXO_4_Z (A: Li, Na; M: group 2, 3, 4, 13 elements; X: group 14, 15, 16 elements; Z: F, Cl, Br, I) were targeted for the ${E}_{b}$-based solid electrolyte screening. The model crystal structure (*P* $\bar{1}$) is shown in Fig. 1a with the host framework comprising with MO_4_Z_2_ octahedra (M 1*a*, 1*h*; O 2*i*) and XO_4_ tetrahedra (X 2*i*; O 2*i*). The MO_4_Z_2_ octahedra are corner-linked together at their *trans*-Z atoms to form chains along [111]. Each oxygen atoms from these chains are in turn shared with X atoms which then assumes a tetrahedral environment. Site splitting occurs for the A atoms (2*i*). Overall, the search space includes LiMXO_4_F dataset taken from our previous work^13^ and newly calculated datasets for LiMXO_4_(Cl/Br/I) and NaMXO_4_(F/Cl/Br/I). Although there are different local A cation pathways in the tavorite structure, our previous calculations determined that its ionic conduction is anisotropic, with the dominant transport pathway being facile in one major cell direction^13^. This conduction pass is defined by a series of local site-to-site jump environments, each sandwiched between two MO_4_Z_2_ octahedra. Hence, ${E}_{b}$ sampling by NEB method was carried out only at the characteristic local pathway bottleneck, as shown in Fig. 1b in asterisk.

### DFT-*E*_*b*_ dataset

**Figure 2:**
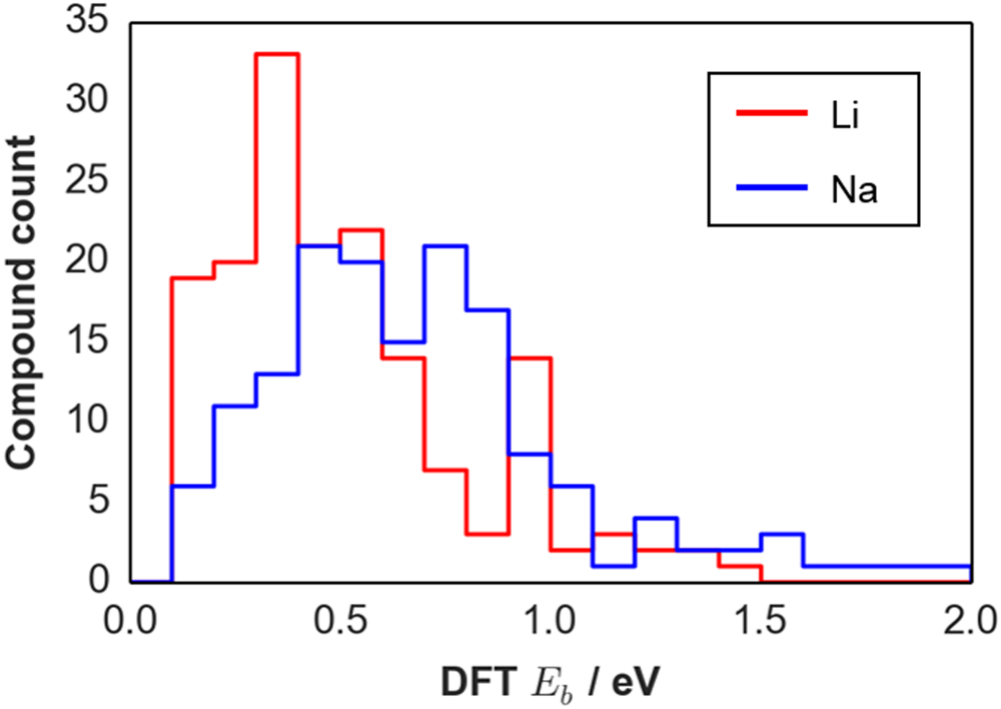
Sample distributions for the *DFT-*${E}_{b}$ datasets prepared for the BO-driven search of tavorite AMXO_4_Z solid electrolytes. There are 163 and 154 DFT-${E}_{b}$ samples contained in the Li and Na dataset, respectively (see Supplementary Table S2 for the actual values).

Figure 2 shows the distribution of Li and Na DFT-${E}_{b}$ datasets (a total of 318 samples) that were prepared in advance for the BO-driven search. We note that although the dataset size may not be large enough for practical material discovery, it should be sufficient enough (considering the heavy computation cost of DFT approach for kinetics-related properties) for evaluating how fast BO-driven search can find the best or nearly-best one in a quinary system. Differences in the sample distribution of the two datasets are revealed by estimating their sample statistics such as maximum ${E}_{b}$ (${E}_{b,max}$), median ${E}_{b}$ (${\tilde{E}}_{b}$), and skewness (${\hat{\alpha }}_{3}$) and kurtosis (${\hat{\alpha }}_{4}$): quantities are {${E}_{b,max}$ = 1.424 eV, ${\tilde{E}}_{b}$ = 0.448 eV, ${\hat{\alpha }}_{3}$ = 0.960, ${\hat{\alpha }}_{4}$ = 0.488} and {${E}_{b,max}$ = 1.965 eV, ${\tilde{E}}_{b}$ = 0.661 eV, ${\hat{\alpha }}_{3}$ = 1.111, ${\hat{\alpha }}_{4}$ = 1.594} for Li and Na, respectively. This comparison clearly shows that the Na case has a broader range and more samples with large ${E}_{b}$ which could mainly stem from the larger atomic mass and ionic radius of Na (${r}_{N{a}^{+}}$ = 1.02 Å vs. ${r}_{L{i}^{+}}$ = 0.76 Å for an octahedral environment)^44^. On another note, both distributions are positively skewed (${\hat{\alpha }}_{3}$ > 0) but with the Na case having a heavier tail towards large ${E}_{b}$ values (${\hat{\alpha }}_{4,Na}$ > ${\hat{\alpha }}_{4,Li}$). These datasets should provide a more stringent performance check for the BO-driven search since random search can favorably sample in the low-${E}_{b}$ density region. The optimal compounds (${{\boldsymbol{x}}}_{{\boldsymbol{\ast }}}$) in the Li and Na datasets are LiScSbO_4_I (${E}_{b}$ = 0.104 eV) and NaErAsO_4_Cl (${E}_{b}$ = 0.116 eV), respectively; both are still unreported compounds.

### BO-driven DFT-*E*_*b*_ search workflow

**Figure 3:**
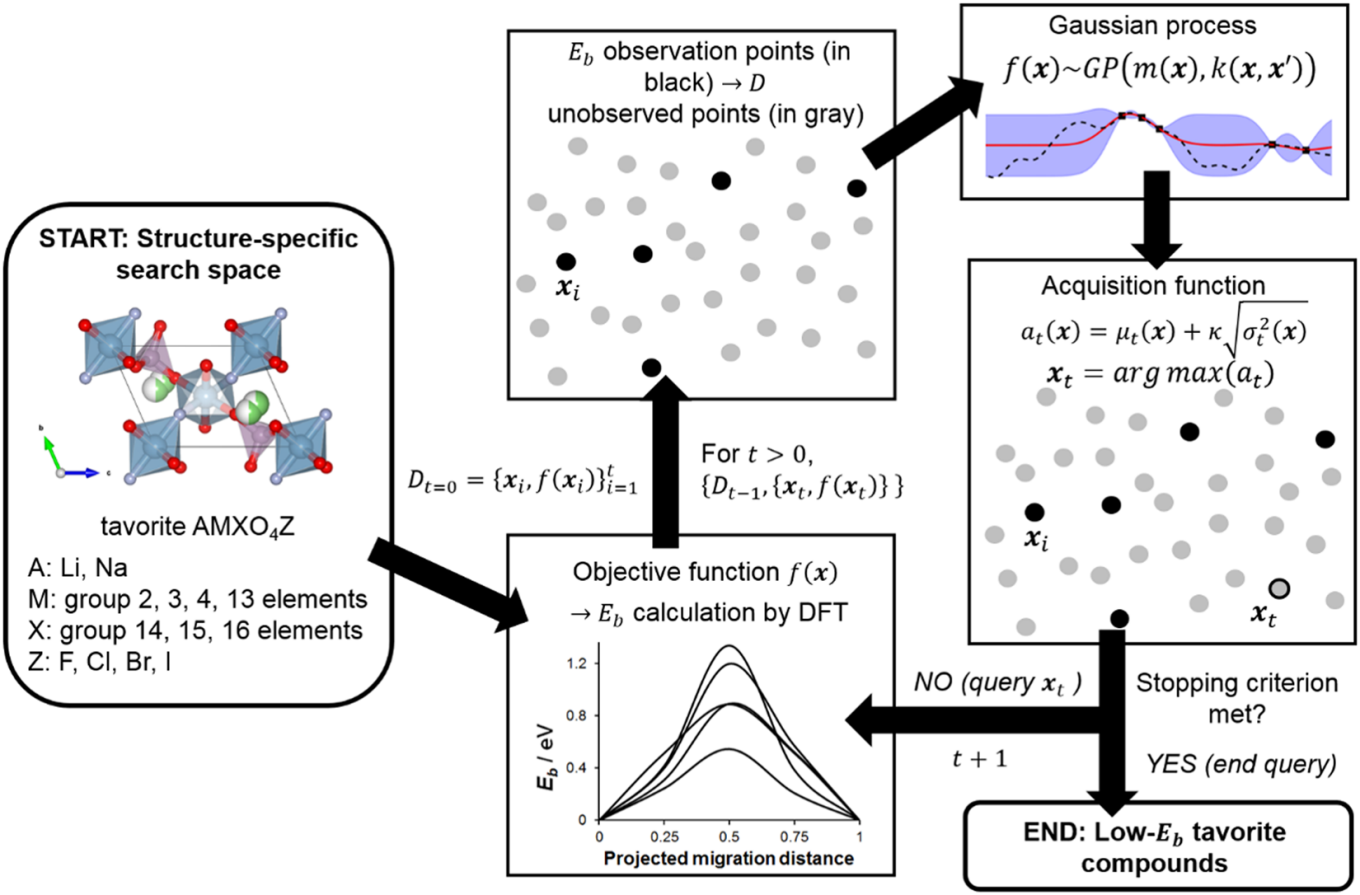
Schematic workflow for the proposed BO-driven DFT-${E}_{b}$ search for tavorite AMXO_4_Z compounds.

Figure 3 shows the schematic workflow for the ${E}_{b}$-based BO-driven search within the AMXO_4_Z tavorite search space. At first, the search space of compounds is populated by various ionic substitutions at the A, M, X, and Z sites. Next, $t$ = 5 initial randomly picked compounds (${\{{{\boldsymbol{x}}}_{i}\}}_{i=1}^{t=5}$) are sampled for DFT-${E}_{b}$ for training the Gaussian Process $GP$ model. The model posterior then provides the predictive mean function ${\mu }_{t}({\boldsymbol{x}})$ and a predictive variance function ${\sigma }_{t}^{2}({\boldsymbol{x}})$ which then defines the acquisition function $a({\boldsymbol{x}})$. Maximizing $a({\boldsymbol{x}})$ then enables for deciding the next query compound ${{\boldsymbol{x}}}_{t}$ to be evaluated for DFT-${E}_{b}$. The sequence is continued until a user-defined number of evaluations or stopping criterion is achieved. In this work, the number of function evaluations was set equal to the number of test data samples.

### Performance evaluation of BO approach

**Figure 4:**
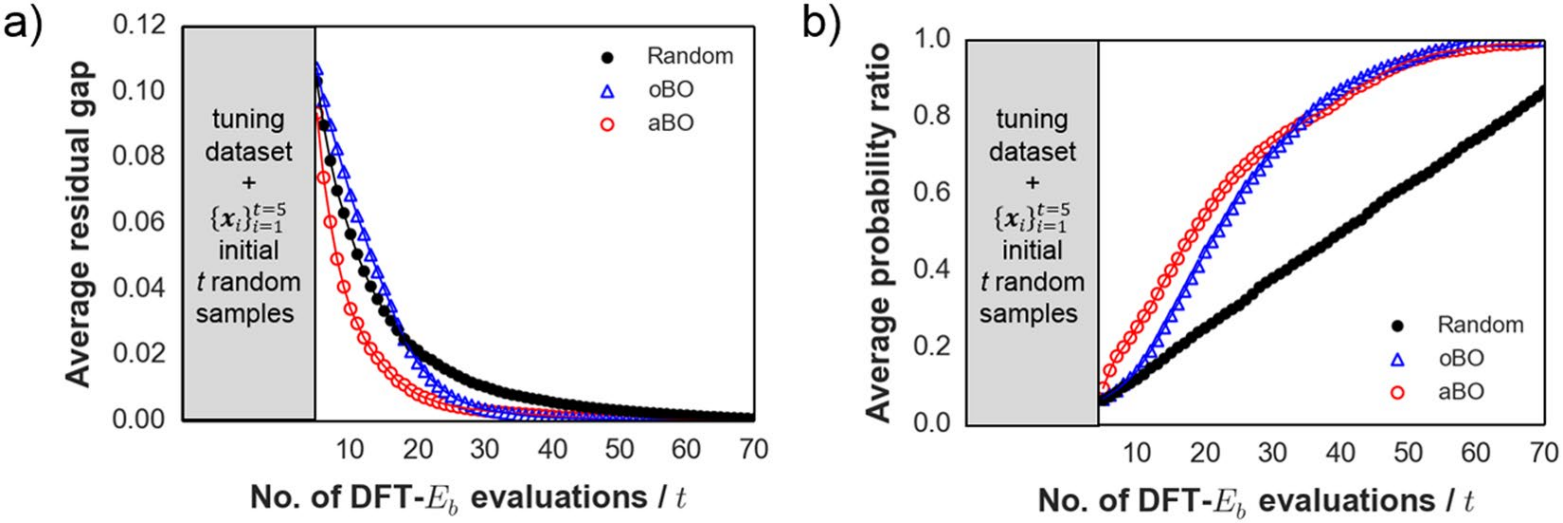
(**a**,**b**) Performance comparison for additive BO (aBO), ordinary (oBO), and random search using the Li-tavorite dataset (averaged from 1000 trials). Horizontal axes for both denote the sequential number of DFT-${E}_{b}$ evaluations $t$. The vertical axis in a) shows the residual gap at step $t$ between optimal $f({{\boldsymbol{x}}}_{{\boldsymbol{\ast }}})$ and the prior best solution ($ma{x}_{{{\boldsymbol{x}}}_{1:t}}\,f({{\boldsymbol{x}}}_{t})$). The vertical axis in (**b**) shows the average probability ratio of discovering the optimal Li-tavorite compound ${{\boldsymbol{x}}}_{{\boldsymbol{\ast }}}$. Note that both additive BO (aBO) and ordinary BO (oBO) search methods here used the tuned hyperparameters from half of the Li dataset excluded for search performance comparison (gray area).

In this paper, the additive BO model is labeled as aBO while the ordinary BO model is labeled as oBO. Figure 4a shows the efficiency of the three search methods for minimizing the residual gap at each evaluation step $t$ between optimal $f({{\boldsymbol{x}}}_{{\boldsymbol{\ast }}})$ and the current best solution ($ma{x}_{{{\boldsymbol{x}}}_{1:t}}f({{\boldsymbol{x}}}_{t})$) for the Li test data. In the high uncertainty regime ($t$ < 20) of the simulated search (i.e., high ${\sigma }^{2}$ since majority of test data compounds are still unobserved), aBO shows the best performance. Meanwhile, oBO performs slightly poorer than random search but when $t$ > 20, it starts to outperform. This behavior for oBO especially at the early stage of the search can be explained by its kernel complexity and the skewed ${E}_{b}$ distribution (see Fig. 3). It should be emphasized though that the nature of the distribution for $f({\boldsymbol{x}})$ is usually not known in advance but incidentally even with a non-normal distribution the BO approach is still generally efficient in querying for low-${E}_{b}$ compounds. Figure 4b shows an alternative performance comparison analysis which is based on the probability ratio of discovering the optimal Li-tavorite compound ${{\boldsymbol{x}}}_{{\boldsymbol{\ast }}}$ as the number of observations $t$ increases (again, as averaged over 1000 trials). The plot indicates that at $t$ = 35, there is already ~80% probability of discovering ${{\boldsymbol{x}}}_{{\boldsymbol{\ast }}}$ for both BO-driven searches.

**Figure 5:**
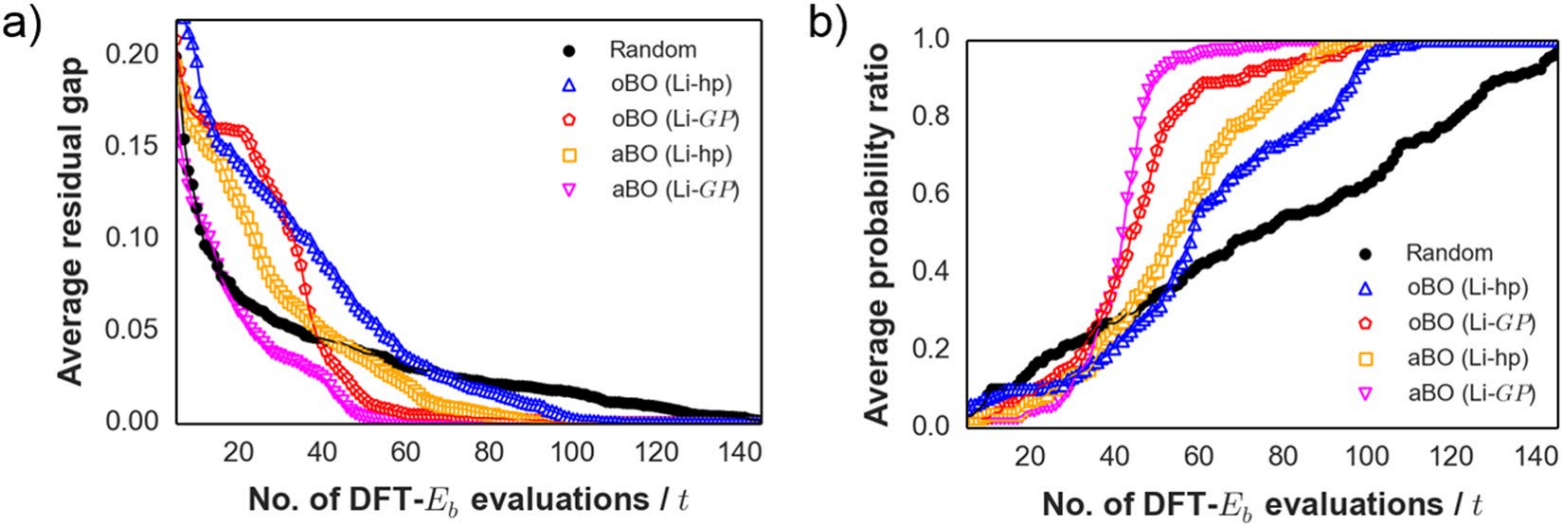
(**a**,**b**) Performance comparison for additive BO (aBO), ordinary BO (oBO), and random search using Na dataset (averaged from 50 trials). Two transfer settings for BO were used: transferred hyperparameters only (Li-hp) and with both transferred hyperparameters and posterior $GP$ (Li-$\,GP$) from Li dataset for BO. Horizontal axes for both denote the number of $f({\boldsymbol{x}})$ evaluations for DFT-${E}_{b}$. The vertical axis in a) denotes the residual gap at each evaluation step $t$ between optimal $f({{\boldsymbol{x}}}_{{\boldsymbol{\ast }}})$ and the prior best solution ($ma{x}_{{{\boldsymbol{x}}}_{1:t}}f({{\boldsymbol{x}}}_{t})$). The vertical axis in (**b**) denotes the percentage ratio of discovering the optimal Na-tavorite compound ${{\boldsymbol{x}}}_{{\boldsymbol{\ast }}}$.

Figure 5a shows the search performance of the 3 methods, for the Na dataset: random search, oBO search, aBO search. Two transfer settings for BO were used: transferred hyperparameters only (Li-hp) and with both transferred hyperparameters and posterior $GP$ (Li-$\,GP$) from Li dataset for BO. The plots are averaged over 50 trials for a search space of 154-compounds test data. For $t$ < 40, oBO is overall performing poorer than random search and aBO regardless of the inherited model settings. This can be primarily explained in a similar fashion as with the Li case, that is, from the viewpoint of kernel complexity and high estimation error when the number of unobserved compounds is still high. Out of the 5 tested models, Li-$GP$ aBO gains a clear advantage over random search for $t$ > 20. These results validate the use of model transfer and demonstrate the predictive power of the trained $GP$ model from the Li dataset for the Na dataset. Additionally shown in Fig. 5b is the probability ratio of finding the optimal Na-tavorite compound ${{\boldsymbol{x}}}_{{\boldsymbol{\ast }}}$. At $t$ = 50 evaluation steps (i.e., ~30% of the search space observed), Li-$GP$ aBO and Li-$GP$ oBO can find ${{\boldsymbol{x}}}_{{\boldsymbol{\ast }}}$ ~90% and ~80% of the time, respectively.

Na and Li ionic conductivity (or Li and Na ion migration energy) are inherently different properties and normally cannot be optimized simultaneously. However, with the systematic approach of knowledge transfer such as in the problem setting (i.e., from Li to Na system), we demonstrated that indeed we can efficiently optimize and find the optimal compound(s) better than random method.

**Figure 6:**
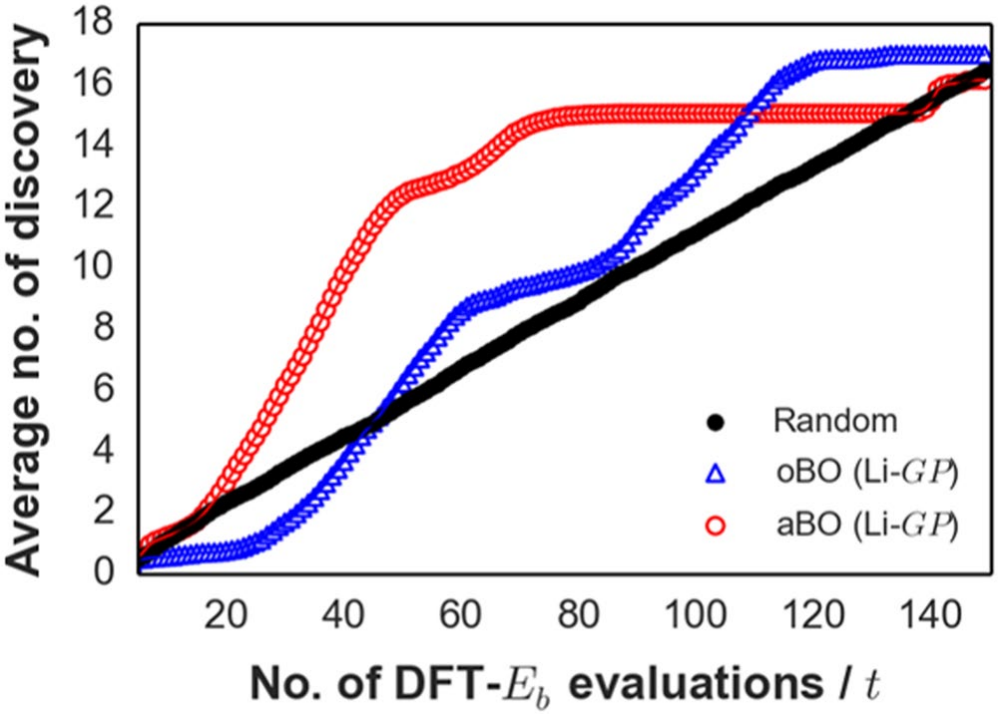
Average number of discovered Na-tavorite compounds with ${E}_{b}$ < 0.3 eV vs. number of DFT evaluations.

The goal of the BO-driven search can be modified so that compounds that satisfy a cutoff value are explicitly searched, in contrast with just finding the single most optimal compound ${{\boldsymbol{x}}}_{{\boldsymbol{\ast }}}$. To demonstrate this, we used the Li-$GP$ transfer model setting and set a criterion of ${E}_{b}$ < 0.3 eV, referred from perovskite Li_0.34_La_0.51_TiO_2.94_ solid electrolyte^45^. The Na dataset was used for performance check and results are displayed in Fig. 6. The vertical axis represents the average number of desired compounds found, which for the Na dataset, would be 17 total compounds meeting the cutoff. For $t$ < 50 (~30% search space coverage), aBO found twice the number of desired compounds than oBO and Random search, discovering ~73% (12.40 compounds) as compared to ~37% (6.26 compounds) and ~33% (5.54 compounds), respectively. However, we note here that aBO failed to find the remaining compounds with ${E}_{b}$ < 0.3 eV even up to ~80% search space coverage ($t$ = 130). This issue is due to the method trading off some of its predictive accuracy for model flexibility. Still, aBO demonstrates its remarkable performance and suitability for large-scale material screening tasks, given that the search is prioritized on maximizing search hits for desired compounds with as few number of DFT-${E}_{b}$ calls as possible.

### Descriptor contribution towards *E*_*b*_ prediction

**Figure 7:**
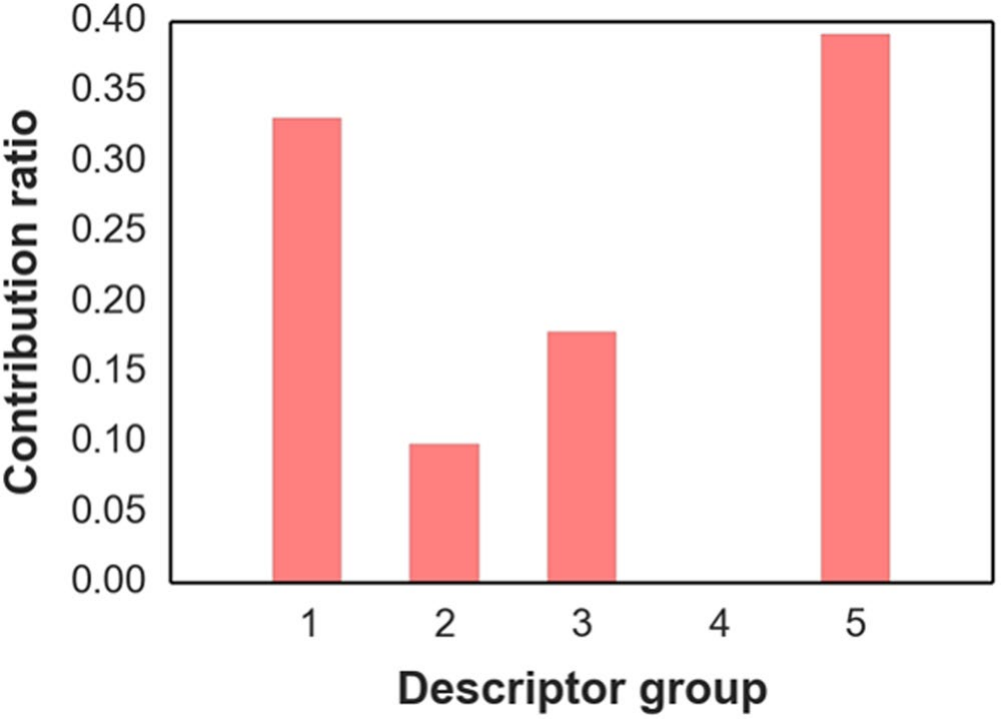
Descriptor group contributions toward ${E}_{b}$ prediction as based from Li-$GP$ aBO model. Vertical axis shows the ratio related to the covariance scale ${\sigma }_{f}^{2}$ of each descriptor group as determined by marginal likelihood maximization.

Another advantage of additive Bayesian optimization is that the importance of each group of descriptors can be easily interpreted. Figure 7 shows the contributions of descriptor groupings for Li-$GP$ aBO towards ${E}_{b}$ prediction. The degree of contribution was calculated by taking the normalized ratio of the covariance amplitude ${\sigma }_{f}^{2}$ for each groups. The two main contributions came from descriptor groups related to the RDF features (g5) and lattice cell features (g1). Meanwhile, inter-polyhedron features (g4) does not contribute and thus could be removed, reducing model complexity from $M$ = 5 down to $M$ = 4 terms. This non-contribution of inter-polyhedron features may be explained by their redundancy since the interatomic-based information contained in them could have been well-expressed already or have been better expressed by RDF features (g5). RDF features, on the other hand, are determined here as effective descriptors for the prediction of ${E}_{b}$ with an inherently structure-independent nature, making it directly applicable for material search/screening tasks with multiple structure types.

To investigate the characteristics of the descriptor values among different compounds, we analyzed the data distribution of some descriptors. We used g1 descriptors (lattice cell features) which were determined automatically by the present BO method as significantly contributing towards ${E}_{b}$ prediction (see Supplementary Fig. 4). We observed that for g1 descriptors, there are different distribution shapes, modalities (unimodal, multi-modal) and degree of skewness for the distribution of values which are indicative of variability and variety in the captured information. In addition, the ranges of each descriptor distributions (see Supplementary Table 5) indicate a varying degree of closeness of values among compounds. Nevertheless, g1 descriptors may be generally regarded as sufficiently differentiating for tavorite compounds. As an example, we examined descriptor df which represents the path bottleneck size for the migrating ion. A value of 0.707 Å (minimum among compounds) would make it geometrically unfavorable for Li ion to pass through (Li ionic radius is 0.76 Å in octahedral coordination, as in the tavorite structure), and much more unfavorable for Na ion (1.02 Å)^44^. Meanwhile, a value of 2.240 Å (maximum among compounds) means both Li and Na ion can pass through geometrically.

Based from above importance analysis on descriptor group contributions, we have shown that our chosen set of descriptors and the strategy of grouping them in their natural groups to define sub-kernel spaces for the BO method is indeed an effective approach for navigating the ion migration energy landscape of the tavorite AMXO_4_Z search space.

### Post-processing of *E*_*b*_-screened tavorite compounds

In an actual material screening task, compounds of interest are usually not only evaluated against a single property but also against other metrics. For example, screened compounds after simulated BO can be further narrowed down by thermodynamic stability criterion to assess whether they can be synthesized by experiment or not. For this purpose, we carried out DFT phase stability calculations based on the convex hull approach and aided by the pymatgen library^46,47^. Briefly, the thermodynamic stability energy (${E}_{d}$) of a given compound was checked against all possible linear combinations of competing phases found in the Materials Project (MP) database^9^. A compound phase may then fall under three cases: (i) ${E}_{d}$ = 0, the compound is predicted to be at the thermodynamic ground state, (ii) ${E}_{d}$> 0, there is a driving force for decomposition, and (iii) for ${E}_{d}$ ≈ 0, a compound is metastable and may be stabilized by appropriate synthesis condition or high kinetic barriers^5^. Based from this classification and from previous empirical results for DFT formation energies, a value of 0.1 eV/atom was chosen as a reasonable upper limit for stability and metastability^5,8^.

**Figure 8:**
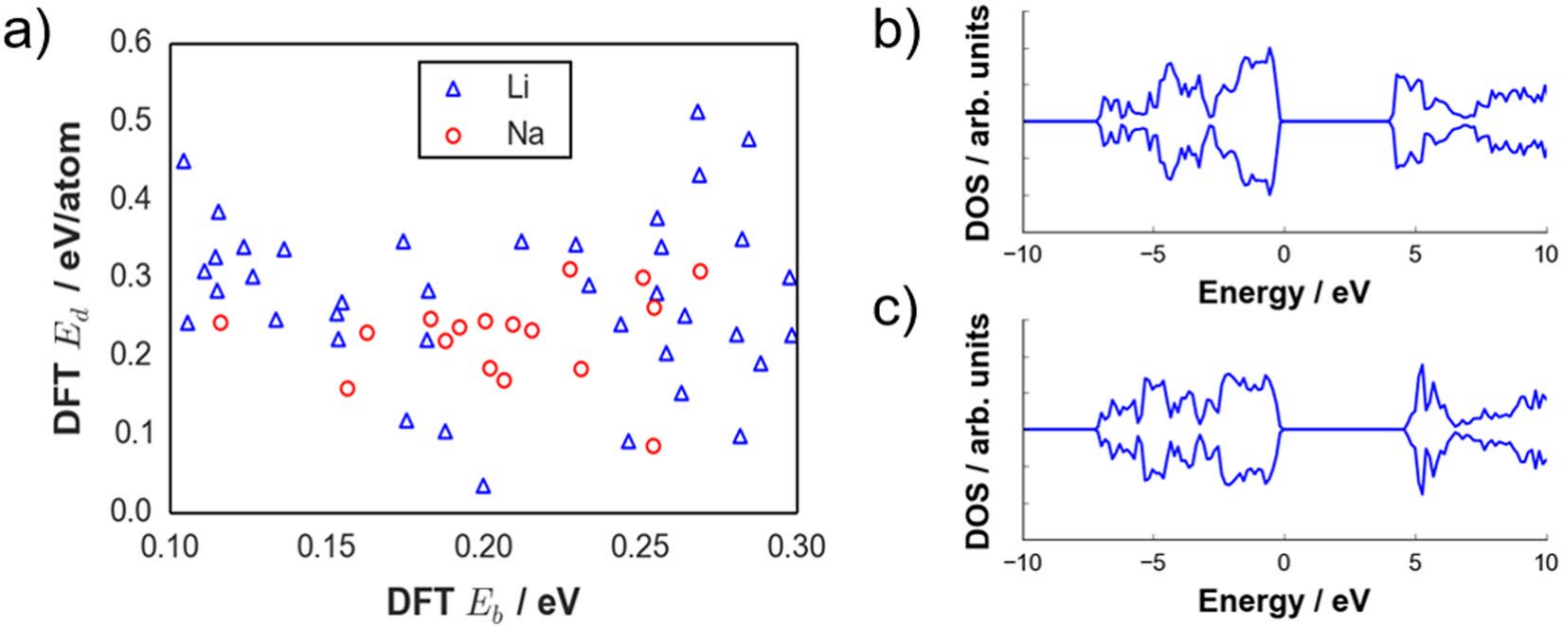
(**a**) DFT-calculated thermodynamic stability energy (${E}_{d}$) of tavorite compounds with ${E}_{b}$ < 0.3 eV. Total density of state of screened representative compounds that passed both ${E}_{b}$ and ${E}_{d}$ cutoffs: (**b**) LiZrGeO_4_F and (**c**) NaHfSiO_4_F.

For the Li-tavorite search space, 20 compounds met the ${E}_{d}$ cutoff. Two of these are recorded in ICSD, LiMgSO_4_F (${E}_{d}$ = 0.034 eV/atom) and LiAlPO_4_F (${E}_{d}$ = 0.016 eV/atom), while the rest are hypothetical compounds that are predicted to be experimentally synthesizable. If both ${E}_{d}$ and ${E}_{b}$ criteria are used, three compounds remained, namely: LiMgSO_4_F (${E}_{b}$ = 0.200 eV, ${E}_{d}$ = 0.035 eV/atom), LiMgSeO_4_Cl (${E}_{b}$ = 0.282 eV, ${E}_{d}$ = 0.098 eV/atom), and LiZrGeO_4_F (${E}_{b}$ = 0.246 eV, ${E}_{d}$ = 0.091 eV/atom). Only LiMgSO_4_F has been characterized so far as a solid electrolyte, whereas the remaining two are new materials. The other database-reported compound is LiAlPO_4_F but it did not pass the ${E}_{b}$ criterion (${E}_{b}$ = 0.550 eV). For the Na-tavorite space, 16 compounds satisfied the ${E}_{d}$ < 0.1 eV/atom condition, all of them are still unreported. Meanwhile, the hypothetical compound NaHfSiO_4_F met both ${E}_{d}$ and ${E}_{b}$ cutoffs (${E}_{b}$ = 0.254 eV, ${E}_{d}$ = 0.085 eV/atom). The ${E}_{d}$ values for the next-tier compounds (in the range 0.3 eV < ${E}_{b}$ < 0.4 eV) are provided in Table S4. Figure 8b,c show the total density of states of LiZrGeO_4_F and NaHfSiO_4_F, with DFT-PBE electronic band gap energies determined to be 4.177 and 4.876 eV, respectively. These values are comparable with other known candidate solid electrolytes such as garnet Li_7_La_3_Zr_2_O_12_ (5.79 eV by HSE06) and Li_10_GeP_2_S_12_ (3.6 eV by PBE) which have wide band gap, indicative of being able to meet the requirement for very low electronic conductivity^48,49^. Additional data are provided in Table S3 for DFT-optimized structural information. The ionothermal synthesis approach would be one of the possible routes for preparing the two new compounds, as demonstrated for already known ones such as tavorite LiMgSO_4_F^37^, and structure-isotopic compounds such as LiFeSO_4_F^36^, LiFePO_4_F^50^, and LiTiPO_4_F^38,50^.

From above results, we have shown that the present DFT-coupled Bayesian optimization approach with additive structure can be applied for quinary systems and when an initial crystal structure type is provided. However, the need for an input structure means that novel compounds with new crystal structures are unsearchable. Nevertheless, we would like to point out that there is now a rich plethora of structure prototypes that can be accessed from existing databases (for example, ICSD presently contains 9,093 structure prototypes)^51^. On another note, other state-of-the-art material search methods have been reported as well, such as crystal structure prediction (CSP) techniques based on evolutionary algorithm^52^. CSP approaches do not require an input structure (the initial atomic arrangement is usually set randomly) but they need composition and initial cell volume. These techniques are meta-heuristic and utilizes empirical rules to govern the search for ground state materials. CSP techniques need to deal with the curse of dimensionality which means that local or global minima structures becomes harder and harder to find as the number of atoms and/or element type increases^52^. Combining our approach with CSP techniques, for example for quinary systems, would be one interesting direction to pursue related to high-dimensionality material search.

## Conclusion

A Bayesian-driven DFT-based screening for Li and Na ionic conductors with the tavorite structure was demonstrated using ion migration energy $({E}_{b})$ as the search criterion. The BO search method was found to be generally more efficient than random search even under a stringent condition of having a positively skewed ${E}_{b}$ sample distributions. Using the Na dataset, additive BO with a knowledge transfer setting requires only an average of ~30% search space coverage to recover the optimal compound ~90% of the time. Using the same test dataset and with a search criterion of ${E}_{b}$ < 0.3 eV, additive BO also only needs to observe ~30% of the search space on the average to find ~70% of the total desired compounds, this is twice the recovery performance for desired materials of ordinary BO and random search which can only find ~37% and ~33%, respectively. These performances are realized with the use of effective descriptors, particularly RDF features. Overall, additive modeling can be an effective approach for addressing the high-dimensionality issue in BO-based searches.

## Methods

### Chemical search space and crystal structure description

Tavorite-type compounds with a general formula AMXO_4_Z (A: Li, Na; M: group 2, 3, 4, 13 elements; X: group 14, 15, 16 elements; Z: F, Cl, Br, I) were targeted for the ${E}_{b}$-based solid electrolyte screening. We note that the M-X cation pair for group 5 and group 13 elements was not included in this study. Although quinary systems have been reported with group 5 cations (e.g., with Ta^5+^ and Nb^5+^ in another structure type^32,53^), group 5 and 13 pairing is highly unlikely in the tavorite structure. This unlikelihood is explained by the deviation of charge distribution for the group 5 - group 13 cation pairing case which leads to a significant destabilization of the crystal structure. The model crystal structure (*P* $\bar{1}$) is shown in Fig. 1a with the host framework comprising with MO_4_Z_2_ octahedra (M 1*a*, 1 *h*; O 2*i*) and XO_4_ tetrahedra (X 2*i*; O 2*i*). The MO_4_Z_2_ octahedra are corner-linked together at their *trans*-Z atoms to form chains along [111]. Each oxygen atoms from these chains are in turn shared with X atoms which then assumes a tetrahedral environment. Site splitting occurs for the A atoms (2*i*). Overall, the search space includes LiMXO_4_F dataset taken from our previous work (13) and newly calculated datasets for LiMXO_4_(Cl/Br/I) and NaMXO_4_(F/Cl/Br/I).

### DFT calculation settings

The VASP code^54^ was used for DFT modeling which applies the projected augmented wave (PAW) approach^55^. The energy for exchange correlation was described in the generalized gradient approximation (GGA) with Perdew-Burke-Ernzernhof formulation for solids (PBEsol)^56^. The initial coordinate dataset for the tavorite structure was referred from available crystal information file (cif) in the Inorganic Crystal Structure Database (ICSD)^52^. With a unit cell of 16 atoms and a spin-polarized condition, a 500-eV cutoff for kinetic energy and a Monkhorst-Pack kpoint resolution of 5 × 4 × 3 were confirmed to show a total energy convergence of less than 1 meV/formula unit (fu). Database-unreported tavorite compounds were calculated using the available experimental cif data as template. The calculation for static atomic charges was based from Bader method^57^. For the dynamical charges, Born effective charge calculation was carried out^58^.

The nudged elastic band (NEB) technique was employed to calculate ${E}_{b}\,$^59^. The unit cell was expanded into a 1 × 2 × 2 supercell and over-the-Brillouin-zone numerical integration was performed by Γ-point sampling. With these conditions, we point out that most of the compounds especially those in the low ${E}_{b}$ region were converged to less than 10 meV/fu (with a few compounds with ${E}_{b}$ > 1.5 eV converged to less than 30 meV/fu). After structure optimization on the initial and final state supercell models containing a single A vacancy, seven images in between for the migrating A cation were constructed by linear interpolation. The value of ${E}_{b}$ was then calculated according to the formula:

1 $${E}_{b}={E}_{max}-{E}_{min}$$

where ${E}_{max}$ and ${E}_{min}$ are the maximum and minimum supercell image energies, respectively, along the migration pathway.

### Material descriptor formulation, formulation of DFT-*E*_*b*_-based search/screening driven by BO

Candidate material descriptors were extracted from the DFT-optimized crystal structures, their description is available in Supplementary Table S1 and Supplementary Figure 1. The resulting initial domain size of the feature space has a total of 348 descriptors. Details on the construction of additive Bayesian model are provided as well in Supplementary Information section.

## Electronic supplementary material

Supplementary Information
