# Supplementary material for: Bayesian-Driven First-Principles Calculations for Accelerating Exploration of Fast Ion Conductors for Rechargeable Battery Application
Source: Sci Rep. 2018 Apr 11;8:5845. doi: 10.1038/s41598-018-23852-y (PMC5895636; doi:10.1038/s41598-018-23852-y)
Supplement: Supplementary file 1 — Supplementary Information [file 41598_2018_23852_MOESM1_ESM.docx]

**Supplementary Information**

Bayesian-Driven First-Principles Calculations for Accelerating Exploration of Fast Ion Conductors for Rechargeable Battery Application

Randy Jalem^a,b,c,1^, Kenta Kanamori^d^, Ichiro Takeuchi^c,d^, Masanobu Nakayama^b,c,e,f^ Hisatsugu Yamasaki^g^_,_ and Toshiya Saito^g^

^a^ Japan Science and Technology Agency (JST), PRESTO, 4-1-8 Honcho Kawaguchi, Saitama 332-0012, Japan

^b^ National Institute for Materials Science – Global Research Center for Environment and Energy based on Nanomaterials Science (NIMS-GREEN), 1-1 Namiki, Tsukuba, Ibaraki 305-0044, Japan

^c^ National Institute for Materials Science – “Materials research by Information Integration” Initiative (NIMS-Mi^2^i), 1-2-1 Sengen, Tsukuba-city Ibaraki 305-0047, Japan

^d^ Department of Computer Science, Nagoya Institute of Technology, Gokiso-cho, Showa-ku, Nagoya, Aichi, 466-8555, Japan

^e^ Department of Advanced Ceramics, Nagoya Institute of Technology, Gokiso-cho, Showa-ku, Nagoya, Aichi 466-8555, Japan

^f^ Elements Strategy Initiative for Catalysts and Batteries, Kyoto University, f1-30 Goryo-Ohara, Nishikyo-ku, Kyoto 615-8245, Japan

^g^ Battery Material Engineering & Research Div., Toyota Motor Corporation, 1200, Mishuku, Susono, Shizuoka 410-1193, Japan

**Abstract**

Safe and robust batteries are urgently requested today for power sources of electric vehicles. Thus, a growing interest has been noted for fabricating those with solid electrolytes. Materials search by density functional theory (DFT) methods offers great promise for finding new solid electrolytes but the evaluation is known to be computationally expensive, particularly on ion migration property. In this work, we proposed a Bayesian-optimization-driven DFT-based approach to efficiently screen for compounds with low ion migration energies ($E_{b})$. We demonstrated this on 318 tavorite-type Li- and Na-containing compounds. We found that the scheme only requires ~ 30% of the total DFT-$E_{b}$ evaluations on the average to recover the optimal compound ~ 90% of the time. Its recovery performance for desired compounds in the tavorite search space is ~ 2x more than random search (i.e., for $E_{b}$ < 0.3 eV). Our approach offers a promising way for addressing computational bottlenecks in large-scale material screening for fast ionic conductors.

**Formulation of DFT-**$\boldsymbol{E}_{\boldsymbol{b}}$**-based search/screening driven by BO.** In an ordinary BO setting with one kernel function that is rather smooth, convergence is expected to be very slow on complex functions, particularly in the case of moderate-to-high dimensionality (Ref. 36-38 of main text). Since the search space of interest in this study is for quinary systems, we expect that the unknown objective function for $E_{b}$ (a kinetics-related property in solids) to be also complex. Noting that the choice of appropriate descriptors for such a complex system is non-trivial, one would usually end up with using more and more features to distinguish materials, leading to a high-dimensional descriptor vector for input material representation. In this work, we prepared 348 initial candidate descriptors that can be extracted cost-effectively from DFT-optimized structures. To address the issue on high dimensionality, we clustered the candidate descriptors into their natural grouping (i.e., geometric, electronic, nearest-neighbor atom/ion information, etc.), and each grouping is utilized to make submodels that individually capture part of the information of the target property $E_{b}$, generating in the end an additive-based overall model (Ref. 39 of main text).

Two DFT-$E_{b}$ tavorite datasets were prepared: 163 and 154 compounds with Li and Na, respectively, as the migrating species within the tavorite host framework (see Supplementary Table 2). The costly objective function $f\left( \boldsymbol{x} \right)$ is the DFT-calculated $E_{b}$ and where $\boldsymbol{x}$ is the multi-dimensional vector which is constructed from various material descriptors. Since low-$E_{b}$ compounds are sought, the optimization scheme is set as the maximization of the negative of $E_{b}$ ($argmax\left( -E_{b} \right)$). Detailed descriptions for BO, Gaussian Process ($GP$), additive BO are provided in the next section. Three search strategies were compared: BO with additive model (aBO), BO with ordinary model (oBO), and random search. For the $GP$ component, a constant mean function $m\left( \boldsymbol{x} \right)$ and an exponential kernel function $k\left( \boldsymbol{x},\boldsymbol{x}' \right)$ specifying the covariance between $\boldsymbol{x}$ and $\boldsymbol{x}’$ (i.e., between two compounds) were used. The acquisition function $a\left( \boldsymbol{x} \right)$ was constructed based on the Upper Confidence Bound (UCB) formalism, with $\kappa$ = 2.0 which equates to 95% confidence bound (See Acquisition Function section below). For aBO, 5 descriptor grouping was made according to the type of information contained in the extracted descriptor: lattice cell features, electronic structure features, intra-polyhedron features, inter-polyhedron features, and atomic radial distribution function (RDF) features (see Table S1) (Ref. 13 of main text). This grouping decomposes the objective function into $M$ = 5 terms:

$f\left( \boldsymbol{x} \right)=\sum_{i=1}^{M=5} f^{\left( i \right)}\left( \boldsymbol{x}^{\left( i \right)} \right)$ (2)

Half of initial $E_{b}$ dataset for Li was used exclusively as tuning data for the hyperparameters and for determining the mean function, whilst remaining half was used as test data for the Li-space BO search. To achieve statistical significance for performance comparison of the three search strategies, 20 different tuning-test data splittings were made, each with 50 randomly instantiated 5 initial observation points. This combination constitutes a total of 1000 trials for each BO-based search strategy. The same procedure for generating trials was also performed for random search.

Subsequently, the Na-space was used to demonstrate the ability of BO method to transfer knowledge (in this case, the knowledge is from the Li-space). Two transfer settings were considered: setting 1 for inheriting only the tuned hyperparameters from Li-space BO search (this setting is called model Li-hp in this work) and setting 2 for inheriting the hyperparameters from Li-space BO search as well as its posterior which is to be used as a prior for the Na-space BO search (this setting is called model Li-$GP$ in this work). This procedure would allow us to systematically validate model transferability and whether the chosen descriptors are advantageous for $E_{b}$ prediction. The experiment on Na dataset was conducted 50 times for each BO-based search strategy. Again, the same procedure for generating trials was also made for random search.

We expect our approach to work in other material discovery settings because it is general enough for high dimensions (convergence issue is addressed through feature subgrouping to lower the effective dimension of the machine model) and choice of descriptors is made flexible (note that in the usual case, choosing the appropriate descriptors for complex material properties is often a non-trivial task).

**Bayesian optimization.** Bayesian optimization (BO) is a sequential optimization algorithm for black-box functions whose evaluation cost is very large^1^. It aims to find the optimal solution for

$\boldsymbol{x}_{*}=arg \max_{\boldsymbol{x}\in\boldsymbol{X}} f\left( \boldsymbol{x} \right), \boldsymbol{X}\subset\mathbb{R}^{d}$, (1)

with as few function evaluations as possible. Generally, BO iterates over following two steps: i) modeling $f\left( \boldsymbol{x} \right)$ by a Gaussian process ($GP$) and ii) maximizing an acquisition function to decide the next observation point $\boldsymbol{x}$ (i.e., the next composition) to evaluate.

**Gaussian process.** Gaussian process ($GP$) is a well-known machine learning technique for predicting an objective function by Bayesian inference^2^. In $GP$, we assume that objective function values $f_{1:n}:=\left[ f\left( \boldsymbol{x}_{1} \right),\ldots,f\left( \boldsymbol{x}_{n} \right) \right]^{\top}$ at arbitrary finite $n$ data points $\boldsymbol{x}_{1:n}$ are drawn from Gaussian distribution characterized by mean function $m\left( \boldsymbol{x} \right):\boldsymbol{X}\mathbb{\to R}$ and kernel (or covariance) function $:\boldsymbol{X}\times\boldsymbol{X}\mathbb{\to R}$.

$f_{1:n}|\boldsymbol{x}_{1:n}\mathcal{\sim N}\left( m_{1:n},\boldsymbol{K} \right)$, (2)

where $K_{i,j}=k\left( \boldsymbol{x}_{i},\boldsymbol{x}_{j} \right)$ ($i,j\in\left\{ 1,\ldots,n \right\}$).

The kernel function defines the covariance between two data points, $\boldsymbol{x}_{i}$ and $\boldsymbol{x}_{j}$. Its most common form is the squared exponential:

$k\left( \boldsymbol{x},\boldsymbol{x'} \right)=\sigma_{f}^{2}exp\left( -\frac{\left\| \boldsymbol{x}-\boldsymbol{x'} \right\|^{2}}{2l^{2}} \right)$ , (3)

where $\sigma_{f}$ and $l$ are positive hyperparameter values that controls the covariance scale and the sensitivity for the difference between two data points, respectively. In addition to assumption (2), the actual observed value of $f\left( \boldsymbol{x}_{i} \right)$ often has an associated noise:

$y_{i}=f\left( \boldsymbol{x}_{i} \right)+\epsilon_{i}$ (4)

with $\epsilon_{i}\mathcal{\sim N}\left( 0,\sigma_{noise}^{2} \right)$. Consequently, given $n$ training data points $\mathcal{D}_{t}:=\left\{ \boldsymbol{x}_{i},y_{i} \right\}_{i=1}^{t}$, a posterior distribution for $f\left( \boldsymbol{x} \right)$ can be generated as follows:

$f\left( \boldsymbol{x} \right)|y_{1:t}\mathcal{\sim N}\left( \mu_{t}\left( \boldsymbol{x} \right),\sigma_{t}^{2}\left( \boldsymbol{x} \right) \right)$, (5)

$\mu_{t}\left( \boldsymbol{x} \right)=m\left( \boldsymbol{x} \right)+\boldsymbol{k}\left( \boldsymbol{x} \right)^{\top}\left( \boldsymbol{K+}\sigma_{noise}^{2}\boldsymbol{I} \right)^{-1}\left( y_{1:t}\boldsymbol{-}m_{1:t} \right)$, (6)

$\sigma_{t}^{2}\left( \boldsymbol{x} \right)=k\left( \boldsymbol{x},\boldsymbol{x} \right)-\boldsymbol{k}\left( \boldsymbol{x} \right)^{\top}\left( \boldsymbol{K+}\sigma_{noise}^{2}\boldsymbol{I} \right)^{-1}\boldsymbol{k}\left( \boldsymbol{x} \right)$, (7)

where $\boldsymbol{k}\left( \boldsymbol{x} \right)$, $m_{1:t}$, $\mu_{t}\left( \boldsymbol{x} \right)$, and $\sigma_{t}^{2}\left( \boldsymbol{x} \right)$ are the covariance vector among $\boldsymbol{x}$ and $\boldsymbol{x}_{1:t}$, the vector mean function of $\boldsymbol{x}_{1:t}$, the posterior mean, and the posterior variance, respectively. It is straightforward to conclude here that the posterior distribution is also a Gaussian distribution. Moreover, the posterior quantities $\mu_{t}\left( \boldsymbol{x} \right)$ and $\sigma_{t}^{2}\left( \boldsymbol{x} \right)$ show the prediction for $f\left( \boldsymbol{x} \right)$ and its prediction uncertainty, respectively. The prediction accuracy largely depends on the hyperparameters of the kernel function which are determined by maximizing the marginal likelihood^2^.

**Acquisition Function.** In the second step of BO, the next function evaluation point $\boldsymbol{x}_{t}$ is selected by maximizing an acquisition function $a\left( \boldsymbol{x} \right)$:

$\boldsymbol{x}_{t}=arg\max_{\boldsymbol{x}} a\left( \boldsymbol{x} \right)$ (8)

Many works have since been made for designing acquisition functions, one of them is based on the $GP$ Upper Confidence Bound ($GP$-$UCB$)^3^:

$a_{t}\left( \boldsymbol{x} \right)=\mu_{t}\left( \boldsymbol{x} \right)+\kappa\sqrt{\sigma_{t}^{2}\left( \boldsymbol{x} \right)}, \kappa\mathbb{\in R}$ (9)

where $\kappa$ is a weight parameter that controls exploration-exploitation tradeoff.

A schematic example for BO in iterative action in 1d is shown in Supplementary Figure 2.

**Gaussian process (GP) with additive structure.** In many instances, BO encounters difficulty when the feature space $\boldsymbol{X}$ has a very high dimensionality (i.e., the number descriptors is very large), that is, modeling for $f\left( \boldsymbol{x} \right)$ by $GP$ tends to perform poorly (32-34, main text). One way to address this problem is to introduce an additive structure to $\boldsymbol{X}$, decomposing $f\left( \boldsymbol{x} \right)$ into a sum of $M\mathbb{\in N}$ functions (35, main text):

$f\left( \boldsymbol{x} \right)=\sum_{i=1}^{M} f^{\left( i \right)}\left( \boldsymbol{x}^{(i)} \right)$ (10)

where $\boldsymbol{x}^{i}\in\boldsymbol{X}^{i}$ and $i=1, 2, \ldots, M$ are the descriptor groups. The groups are disjoint (i.e., if the vector elements $\boldsymbol{x}$ are treated as a set, $\boldsymbol{x}^{\left( i \right)}\cap\boldsymbol{x}^{\left( j \right)}=\emptyset$). By expressing $f\left( \boldsymbol{x} \right)$ as the sum of functions $f^{\left( i \right)}\left( \boldsymbol{x}^{\left( i \right)} \right)$, the additive model simplifies the feature space. As a consequence, the mean function $m$ and the kernel function $k$ are also each replaced with a summation, with each term coming from the descriptor grouping:

$m\left( \boldsymbol{x} \right)=\sum_{i=1}^{M} m^{\left( i \right)}\left( \boldsymbol{x}^{\left( i \right)} \right)$ (11)

$k\left( \boldsymbol{x},\boldsymbol{x}' \right)=\sum_{i=1}^{M} k^{\left( i \right)}\left( \boldsymbol{x}^{\left( i \right)},{\boldsymbol{x}'}^{\left( i \right)} \right)$ (12)

An intuitive example on the properties of Additive $GP$ can be seen in Supplementary Figure 3. The figure shows an example comparison for calculating $k(\boldsymbol{x}, [0, 0])$ using a normal squared exponential kernel and a kernel function applying additive model with each dimension as a group in the two-dimensional feature space. It is evident here that with the normal squared exponential kernel, positions far from point [0, 0] hardly takes a value, whereas in the additive model the kernel function has a wider range along each feature axis. Meanwhile, Fig. S3b shows the prediction comparison for the test objective function $f\left( \boldsymbol{x} \right)=sin\left( x^{\left( 1 \right)} \right)+cos\left( x^{\left( 2 \right)} \right)$ between GP with additive modeling and normal GP, indicating the efficient modeling performance of the former using the same number of observations.


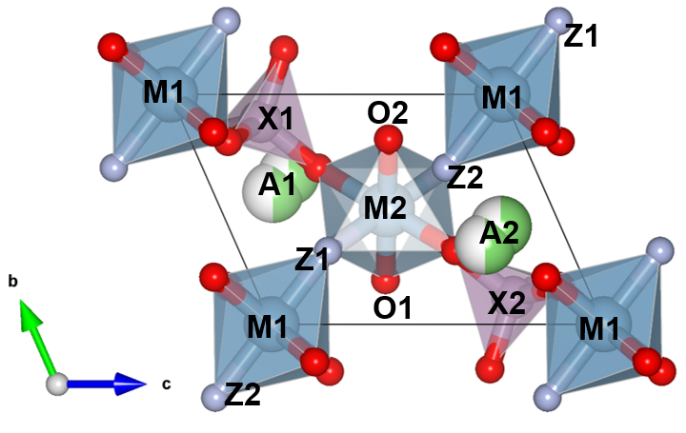


**Supplementary Figure 1.** Visual reference for the extracted inter-polyhedron parameters.


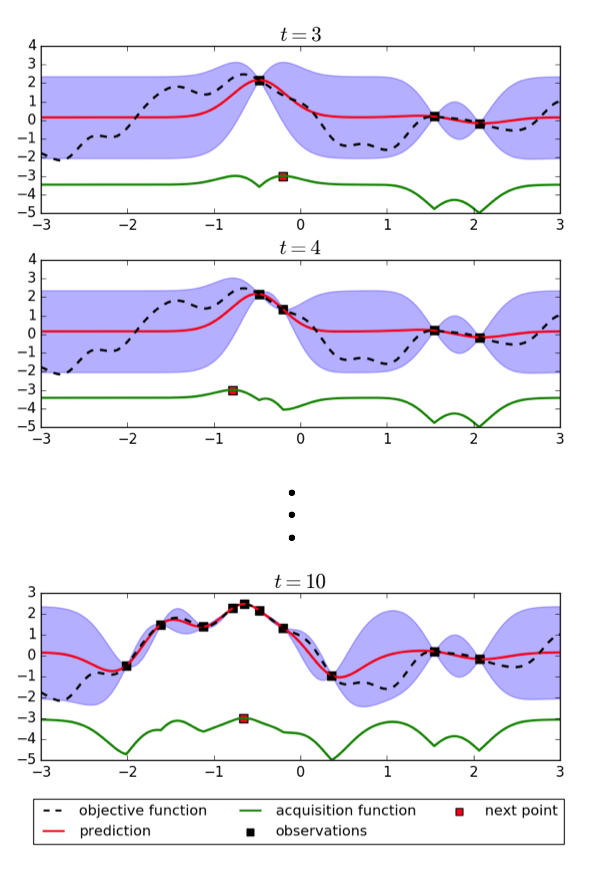


**Supplementary Figure 2.** An example of a 1-d Bayesian optimization process. The horizontal axis, left vertical axis, and right vertical axis represent the feature $\boldsymbol{x}$, the objective function $f\left( \boldsymbol{x} \right)$, and the acquisition function $a\left( \boldsymbol{x} \right)$ (scaling not shown explicitly), respectively. With an increasing number of trials $t$, the estimation uncertainty along $\boldsymbol{x}$ (indicated by the vertical width on the blue area) shrinks progressively.


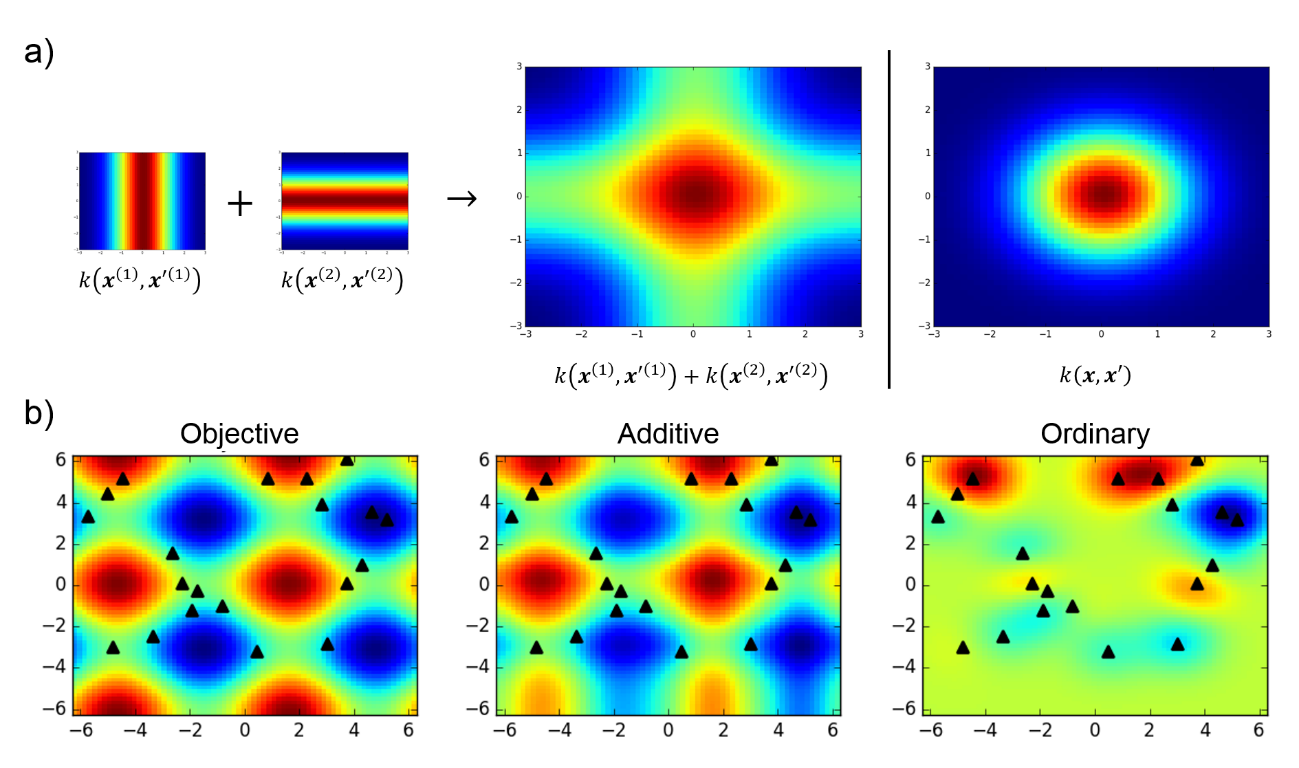


**Supplementary Figure 3.** a) Comparison for $k(\boldsymbol{x}, [0, 0])$ calculation in the two-dimensional feature space between a squared exponential kernel that incorporates additive models with $M = 2$ descriptor groups (left side of the vertical bar) and the normal squared exponential kernel (right side of the vertical bar). b) Comparison between Gaussian process with additive modeling and ordinary Gaussian process. The left subfigure is the test objective function $f\left( \boldsymbol{x} \right)=sin\left( x^{\left( 1 \right)} \right)+cos\left( x^{\left( 2 \right)} \right)$, black triangles represent the observation points (costly to evaluate).


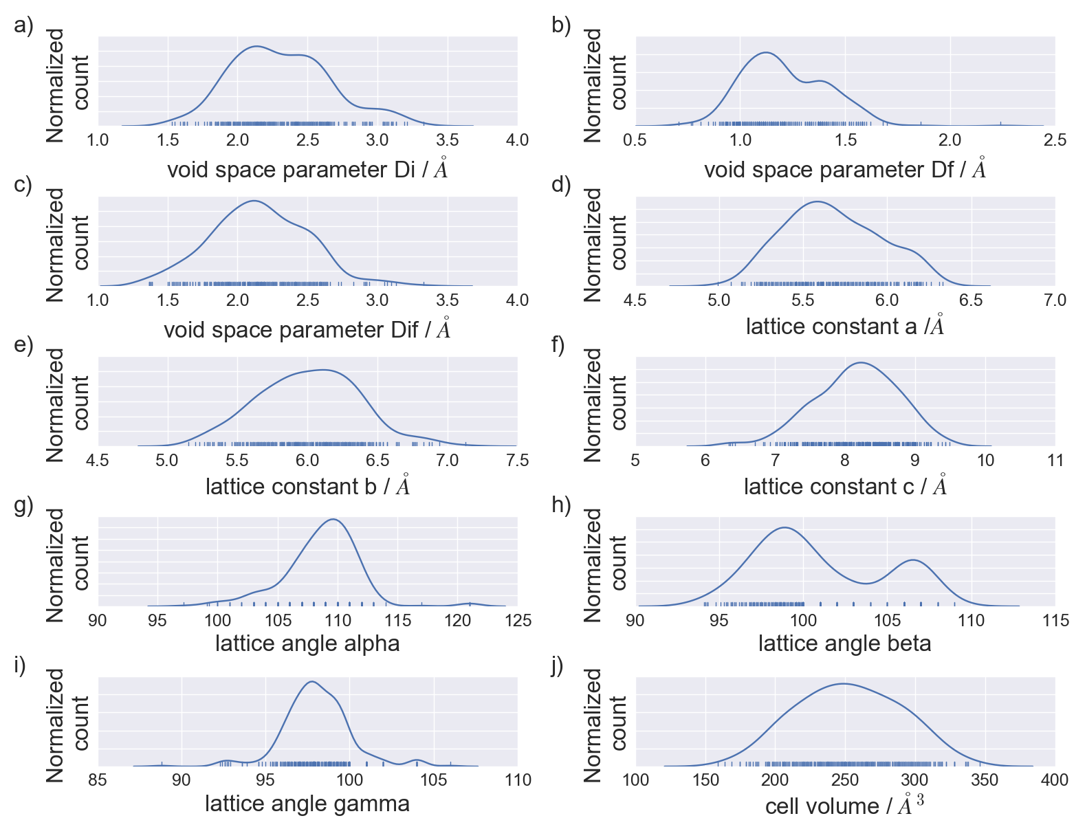


**Supplementary Figure 4.** Normalized distribution plots of group 1 descriptor values. Normalized distribution plots of group 1 descriptor values. Rug lines at the base of the distributions mark the actual descriptor values.

**Supplementary Table S1.** Extract descriptors from DFT-optimized structures.

| Descriptor grouping | Symbol | Description |
| --- | --- | --- |
| Lattice cell features | di | largest included sphere related to the void space after A atom removal^4^ |
|  | df | largest free sphere related to the void space after A atom removal^4^ |
|  | dif | largest included sphere along the free sphere path related to the void space after A atom removal^4^ |
|  | a | Unit cell lattice parameter *a* |
|  | b | Unit cell lattice parameter *b* |
|  | c | Unit cell lattice parameter *c* |
|  | alpha | Lattice angle *α* |
|  | beta | Lattice angle *β* |
|  | gamma | Lattice angle *γ* |
|  | vol | Unit cell volume |
| Electronic structure features | bd1, bd2, …, bd16 | Bader charges of all unit cell atoms (16 atoms) |
|  | bec1, bec2, …, bec16 | Born effective charges from all unit cell atoms (16 atoms) |
| Geometric intra-polyhedron  features | abl1, abl2, …, abl6 | Average bond length in each of unit cell polyedron (two A octahedra, two M octahedra, two X tetrahedra) |
|  | pv1, pv2, …, pv6 | Volume in each unit cell polyhedron (two A octahedra, two M octahedra, two X tetrahedra)^5^ |
|  | di1, di2, …, di6 | Distortion index in each unit cell polyhedron (two A octahedra, two M octahedra, two X tetrahedra)^6^ |
|  | qe1, qe2, …, qe6 | Quadratic elongation in each unit cell polyhedron (two A octahedra, two M octahedra, two X tetrahedra)^7^ |
|  | bav1, bav2, …, bav6 | Bond angle variance in each unit cell polyhedron (two A octahedra, two M octahedra, two X tetrahedra)^7^ |
|  | ecn1, ecn2, …, ecn6 | Effective coordination number in each unit cell polyhedron (two A octahedra, two M octahedra, two X tetrahedra)^8^ |
| Geometric inter-polyhedron features | M1Z2M2 | M1-Z2-M2 angle* |
|  | M2O2A2 | M2-O2-A2 angle* |
|  | M2O2X2 | M2-O2-X2 angle* |
|  | M2Z1M1 | M2-Z1-M1 angle* |
|  | Z1Z2Z1 | MO_4_Z_2_-MO_4_Z_2_ angle with common Z2 atom as vertex* |
|  | A2Z2M2 | A2-Z-2M2 angle* |
|  | A2Z2M1 | A2-Z2-M1 angle* |
|  | M2M2p | Distance from M2 atom to the first nearest neighbor M2 atom of another MO_4_Z_2_ chain* |
|  | M2(1)M2(3) | Distance from M2 atom to the second next nearest neighbor M2 atom of the same MO_4_Z_2_ chain* |
|  | M2-X1 | Distance from M2 atom to the first nearest neighbor X1 atom* |
|  | M2O1A1 | M2-O1-A1 angle* |
|  | M2O1X1 | M2-O1-X1 angle* |
|  | Z2Z1Z2 | Z2-Z1-Z2 angle from within one MO_4_Z_2_ chain* |
|  | A1Z1M1 | A1-Z1-M1 angle* |
|  | A1Z1M2 | A1-Z1-M1 angle* |
|  | M2A1 | Distance from M2 atom to the neighbor A1 atom* |
|  | M2-A2 | Distance from M2 atom to the neighbor A2 atom* |
|  | A1-X1 | Distance from A1 atom to the neighbor X1 atom* |
|  | A2-X2 | Distance from A2 atom to the neighbor X2 atom* |
| Overall radial distribution function (RDF) features | r1, r2, …, r251 | bin features from unbroadened overall RDF histogram (bin size = 0.02 Å, cutoff = 5.02 Å) |

*Visual reference shown in Supplementary figure 1.

**Supplementary Table S2.** Dataset for DFT-calculated Li/Na ion migration energies ($E_{b}$); AMXZ for element name sequence in AMXO_4_Z composition.

| AMXZ | $E_{b}$ / eV | AMXZ | $E_{b}$ / eV | AMXZ | $E_{b}$ / eV | AMXZ | $E_{b}$ / eV |
| --- | --- | --- | --- | --- | --- | --- | --- |
| LiScPF | 0.3843 | LiHoAsF | 0.9732 | NaScPF | 0.6687 | NaAlPBr | 1.5351 |
| LiScPCl | 0.1879 | LiHoAsCl | 0.3822 | NaScPCl | 0.2067 | NaAlAsCl | 0.5229 |
| LiScPBr | 0.3064 | LiHoAsBr | 0.3148 | NaScPBr | 0.3430 | NaAlSbF | 0.5352 |
| LiScPI | 0.7815 | LiHoAsI | 0.2826 | NaScAsF | 0.7280 | NaAlSbCl | 0.5425 |
| LiScAsF | 0.4838 | LiHoSbBr | 0.1235 | NaScAsCl | 0.1566 | NaAlSbBr | 0.4475 |
| LiScAsCl | 0.3753 | LiHoSbI | 0.1364 | NaScSbCl | 0.8744 | NaGaPF | 1.3309 |
| LiScAsBr | 0.2633 | LiErPF | 0.7623 | NaScSbBr | 0.3429 | NaGaPCl | 0.4460 |
| LiScAsI | 0.1745 | LiErPI | 0.5297 | NaYPCl | 0.1879 | NaGaPBr | 0.6985 |
| LiScSbF | 0.5873 | LiErAsF | 0.9969 | NaYAsF | 0.4945 | NaGaAsF | 1.3123 |
| LiScSbCl | 0.1820 | LiErAsCl | 0.4165 | NaYSbF | 0.6614 | NaGaAsCl | 0.4692 |
| LiScSbI | 0.1043 | LiErAsI | 0.2122 | NaYSbCl | 0.4491 | NaGaSbBr | 0.9700 |
| LiYPCl | 0.3386 | LiErSbBr | 0.1110 | NaLaPF | 0.8179 | NaInPCl | 0.3844 |
| LiYPI | 0.1264 | LiTmPF | 0.7031 | NaLaPCl | 0.8011 | NaInPBr | 1.0713 |
| LiYAsF | 1.0754 | LiTmPBr | 0.1056 | NaLaPBr | 0.8281 | NaInAsF | 0.8932 |
| LiYAsCl | 0.4009 | LiTmAsF | 0.9715 | NaLaSbF | 0.9291 | NaInSbF | 1.4956 |
| LiYSbCl | 0.1150 | LiTmAsCl | 0.3832 | NaLaSbCl | 0.5075 | NaInSbCl | 0.5150 |
| LiYSbBr | 0.1145 | LiTmAsBr | 0.2985 | NaLaSbBr | 0.7578 | NaInSbBr | 0.3272 |
| LiLaPCl | 0.6545 | LiTmSbF | 0.9851 | NaCeSbCl | 0.7270 | NaMgSF | 0.8109 |
| LiLaPBr | 0.5549 | LiAlPF | 0.5788 | NaCeSbBr | 0.7149 | NaMgSBr | 0.5401 |
| LiLaPI | 0.4059 | LiAlPCl | 0.3108 | NaPrPCl | 1.0150 | NaMgSeF | 0.9533 |
| LiLaAsF | 1.3049 | LiAlPBr | 0.1338 | NaPrPBr | 0.3583 | NaMgSeCl | 0.6649 |
| LiLaAsCl | 0.6016 | LiAlAsCl | 0.3925 | NaPrAsF | 0.6720 | NaMgSeBr | 0.3509 |
| LiLaAsBr | 0.6121 | LiAlAsBr | 0.2886 | NaPrAsCl | 0.7388 | NaMgTeF | 1.1352 |
| LiLaSbI | 0.2556 | LiAlAsI | 0.1155 | NaPrSbF | 0.6202 | NaMgTeCl | 0.8271 |
| LiCePF | 1.3280 | LiAlSbF | 0.9368 | NaPrSbCl | 0.7092 | NaMgTeBr | 0.6256 |
| LiCePCl | 0.6766 | LiAlSbCl | 0.2809 | NaPrSbBr | 0.6925 | NaCaSF | 0.8112 |
| LiCePBr | 0.5618 | LiGaPF | 0.4839 | NaNdPF | 0.8539 | NaCaSCl | 0.2022 |
| LiCePI | 1.0366 | LiGaPCl | 0.3660 | NaNdPCl | 0.7564 | NaCaSBr | 0.2155 |
| LiCeAsF | 1.2721 | LiGaPBr | 0.2584 | NaNdPBr | 0.8720 | NaCaSeF | 1.2382 |
| LiCeAsCl | 0.5723 | LiGaPI | 0.8566 | NaNdSbF | 1.5710 | NaCaSeCl | 0.3398 |
| LiCeAsBr | 0.5995 | LiGaAsF | 0.6622 | NaNdSbBr | 0.6714 | NaCaSeBr | 0.2312 |
| LiCeAsI | 0.4271 | LiGaAsCl | 0.3957 | NaPmPF | 0.8932 | NaCaTeF | 0.5600 |
| LiCeSbCl | 0.3661 | LiGaAsBr | 0.3349 | NaPmPCl | 0.5258 | NaCaTeCl | 0.2510 |
| LiCeSbBr | 0.4042 | LiGaSbF | 0.8341 | NaPmAsF | 0.5940 | NaCaTeBr | 0.4477 |
| LiCeSbI | 0.7065 | LiGaSbBr | 0.1825 | NaPmAsCl | 0.5717 | NaSrSF | 0.7877 |
| LiPrPF | 0.7254 | LiInPF | 0.3678 | NaPmAsBr | 0.5864 | NaSrSBr | 0.9268 |
| LiPrPCl | 0.5387 | LiInPBr | 1.1282 | NaPmSbCl | 0.4285 | NaSrSeF | 0.5632 |
| LiPrPBr | 0.5707 | LiInAsF | 1.4242 | NaPmSbBr | 0.5895 | NaSrSeCl | 0.9705 |
| LiPrPI | 0.5093 | LiInAsCl | 0.3897 | NaSmPCl | 1.2468 | NaSrSeBr | 1.2167 |
| LiPrAsF | 0.9200 | LiInAsBr | 0.3913 | NaSmAsF | 0.7840 | NaSrTeCl | 0.3074 |
| LiPrAsCl | 0.3616 | LiInSbF | 0.6141 | NaSmAsCl | 1.0571 | NaSrTeBr | 1.0619 |
| LiPrAsBr | 0.4904 | LiInSbI | 0.5506 | NaSmAsBr | 0.9655 | NaBaSF | 1.0066 |
| LiPrAsI | 0.4055 | LiMgSF | 0.2834 | NaSmSbF | 1.4790 | NaBaSCl | 0.9977 |
| LiPrSbF | 0.6142 | LiMgSI | 0.9151 | NaSmSbCl | 0.4181 | NaBaSBr | 0.7767 |
| LiPrSbBr | 0.3532 | LiMgSeI | 0.8775 | NaSmSbBr | 0.6069 | NaBaSeF | 0.9906 |
| LiPrSbI | 0.3382 | LiMgTeBr | 0.1549 | NaGdPF | 0.5688 | NaBaSeCl | 0.4913 |
| LiNdPCl | 0.5548 | LiCaSCl | 0.5719 | NaGdAsCl | 0.6627 | NaBaSeBr | 0.7054 |
| LiNdPBr | 0.5605 | LiCaSeCl | 0.1755 | NaGdAsBr | 1.7551 | NaBaTeCl | 0.8592 |
| LiNdAsF | 1.2783 | LiCaTeCl | 0.1533 | NaGdSbF | 1.9653 | NaTiSiF | 1.2059 |
| LiNdAsBr | 0.4858 | LiSrSF | 0.9953 | NaGdSbCl | 0.4238 | NaTiSiBr | 0.8140 |
| LiNdAsI | 0.6418 | LiSrSCl | 0.4602 | NaGdSbBr | 0.4815 | NaTiGeF | 1.0275 |
| LiNdSbF | 0.6581 | LiSrSBr | 0.3139 | NaTbPF | 0.4571 | NaTiGeCl | 0.3315 |
| LiPmPF | 0.6180 | LiSrSeBr | 0.4744 | NaTbPCl | 0.3134 | NaTiGeBr | 0.2693 |
| LiPmPCl | 0.5590 | LiBaSeF | 0.9500 | NaTbAsF | 0.5465 | NaTiSnF | 0.8085 |
| LiPmPI | 0.4476 | LiBaSeCl | 0.5659 | NaTbAsBr | 1.5603 | NaTiSnCl | 0.6169 |
| LiPmAsBr | 0.4709 | LiBaSeBr | 0.5511 | NaTbSbCl | 0.4443 | NaTiSnBr | 0.5499 |
| LiPmAsI | 0.5798 | LiBaTeBr | 0.4946 | NaDyPCl | 0.2007 | NaZrSiF | 0.7069 |
| LiPmSbF | 0.6463 | LiTiSiF | 0.9966 | NaDyAsF | 0.4953 | NaZrSiBr | 0.7618 |
| LiPmSbBr | 1.1214 | LiTiSiBr | 0.9734 | NaDyAsCl | 0.3276 | NaZrGeF | 0.7916 |
| LiSmPCl | 0.5578 | LiTiGeCl | 0.4492 | NaDySbF | 1.8949 | NaZrGeCl | 0.3646 |
| LiSmPBr | 0.1537 | LiTiGeI | 0.4552 | NaDySbBr | 0.4636 | NaZrGeBr | 0.6950 |
| LiSmPI | 0.4353 | LiTiSnF | 0.7354 | NaHoPF | 0.5539 | NaZrSnF | 0.7661 |
| LiSmAsCl | 0.4051 | LiTiSnI | 0.5444 | NaHoPCl | 0.2095 | NaZrSnCl | 0.6387 |
| LiSmAsI | 0.2690 | LiZrSiF | 0.9054 | NaHoAsCl | 0.1832 | NaZrSnBr | 0.7086 |
| LiSmSbF | 0.7031 | LiZrSiCl | 0.4474 | NaHoAsBr | 0.2277 | NaHfSiF | 0.2543 |
| LiGdPI | 0.3517 | LiZrSiI | 0.6273 | NaHoSbF | 0.6610 | NaHfSiCl | 0.2546 |
| LiGdAsF | 0.9867 | LiZrGeF | 0.2464 | NaHoSbCl | 0.4425 | NaHfSiBr | 0.8880 |
| LiGdAsCl | 0.6493 | LiZrGeCl | 0.3972 | NaHoSbBr | 0.7177 | NaHfGeF | 0.8912 |
| LiGdSbI | 0.1168 | LiZrGeBr | 0.3840 | NaErPF | 0.4477 | NaHfGeCl | 0.3008 |
| LiTbPF | 0.3423 | LiZrSnF | 0.5397 | NaErPCl | 0.1924 | NaHfGeBr | 0.7946 |
| LiTbPCl | 0.3720 | LiZrSnBr | 0.3279 | NaErPBr | 0.5170 | NaHfSnF | 0.7323 |
| LiTbPI | 0.3029 | LiZrSnI | 0.2848 | NaErAsF | 0.5654 | NaHfSnBr | 0.7189 |
| LiTbAsCl | 0.4988 | LiHfSiF | 0.6161 | NaErAsCl | 0.1161 |  |  |
| LiTbAsBr | 0.2439 | LiHfSiCl | 0.3064 | NaErSbCl | 0.4887 |  |  |
| LiTbSbCl | 0.2978 | LiHfSiBr | 0.2644 | NaErSbBr | 0.4410 |  |  |
| LiTbSbBr | 0.2569 | LiHfGeF | 0.5248 | NaTmPBr | 0.8688 |  |  |
| LiDyPF | 0.3207 | LiHfGeBr | 0.3281 | NaTmAsF | 0.5625 |  |  |
| LiDyAsF | 1.1058 | LiHfGeI | 0.3005 | NaTmAsCl | 0.1629 |  |  |
| LiDyAsBr | 0.3374 | LiHfSnF | 0.2554 | NaTmSbF | 0.7070 |  |  |
| LiDyAsI | 0.2296 | LiHfSnCl | 0.3035 | NaTmSbCl | 0.4793 |  |  |
| LiDySbCl | 0.2338 | LiHfSnI | 0.2685 | NaAlPF | 1.6210 |  |  |
| LiHoPF | 0.9203 |  |  | NaAlPCl | 0.4959 |  |  |

**Supplementary Table S3**. DFT-calculated structural information for two representative tavorite compounds satisfying both ion migration energy ($E_{b}$) and thermodynamic stability ($E_{d}$) criteria.

| Parameter | LiZrGeO_4_F | NaHfSiO_4_F |
| --- | --- | --- |
| *a* / Å | 5.5316 | 5.5297 |
| *b* / Å | 5.7018 | 5.5380 |
| *c* / Å | 7.6915 | 7.7386 |
| *α* | 109.718 | 106.215 |
| *β* | 105.875 | 105.856 |
| *γ* | 97.472 | 104.219 |
| Volume / Å^3^ | 212.93 | 205.17 |

**Supplementary Table S4**. DFT-calculated decomposition energy ($E_{d}$ $E_{d}$) for tavorite compounds that have ion migration energy ($E_{b}$ $E_{b}$) values in the range 0.3 eV < $E_{b}$ $E_{b}$ < 0.4 eV; AMXZ for element name sequence in AMXO_4_Z composition.

| AMXZ | $E_{b}$ $E_{d}$ / eV/atom | AMXZ | $E_{b}$ $E_{d}$ / eV/atom |
| --- | --- | --- | --- |
| LiScPF | 0.0306 | NaScPBr | 0.2287 |
| LiScPBr | 0.1854 | NaScSbBr | 0.3270 |
| LiPrSbBr | 0.3970 | NaPrPBr | 0.2721 |
| LiPrSbI | 0.3674 | NaTbPCl | 0.2487 |
| LiTbPCl | 0.1632 | NaDyAsCl | 0.2540 |
| LiTbPI | 0.3496 | NaInPCl | 0.1558 |
| LiHoAsBr | 0.2330 | NaInSbBr | 0.3427 |
| LiAlAsCl | 0.1177 | NaMgSeBr | 0.2061 |
| LiGaPCl | 0.1413 | NaCaSeCl | 0.1312 |
| LiGaAsCl | 0.1177 | NaSrTeCl | 0.3550 |
| LiInPF | 0.0500 | NaTiGeCl | 0.2469 |
| LiInAsCl | 0.1088 | NaHfGeCl | 0.2659 |
| LiInAsBr | 0.1648 |  |  |

**Supplementary Table S5**. Minimum and maximum values of group 1 descriptors (see Supplementary Table 1 for descriptor description).

| Descriptor symbol | (minimum, maximum), ∆ |
| --- | --- |
| di | (1.530 Å, 3.330 Å), |
| df | (0.707 Å, 2.240 Å) |
| dif | (1.370 Å, 3.330 Å) |
| a | (4.990 Å, 6.330 Å) |
| b | (5.150 Å, 7.130 Å) |
| c | (6.340 Å, 9.490 Å) |
| alpha | (97.20, 121.00) |
| beta | (94.10, 109.00) |
| gamma | (88.80, 106.00) |
| vol | (159.00 Å^3^, 346.00 Å^3^) |

**Supplementary References**

1. Shahriari, B., Swersky, K., Wang, Z., Adams, R. P. & de Freitas, N. Taking the human out of the loop: A review of bayesian optimization. *Proceedings of the IEEE 2016* **104**, 148-175 (2016).
2. Rasmussen, C. E. *Gaussian processes for machine learning*, MIT press (2006).
3. Srinivas, N., Krause, A., Kakade, S. M. & Seeger, M. Gaussian process optimization in the bandit setting: No regret and experimental design. *In International Conference on Machine Learning, ICML* (2010).
4. Willems, T. F., Rycroft, C. H., Kazi, M., Meza, J. C. & Haranczyk, M. Algorithms and tools for high-throughput geometry- based analysis of crystalline porous materials. *Micropor. Mesopor. Mat.* **149**, 134-141 (2012).
5. Swanson, D. K. & Peterson, R. C. Polyhedral Volume Calculations. *Can. Mineral.* **18**, 153-156 (1980).
6. Baur, W. H. The geometry of polyhedral distortions. Predictive relationships for the phosphate group. *Acta Crystallogr., Sect. B: Struct. Sci.* **B30**, 1195-1215 (1974).
7. Robinson, K., Gibbs, G. V. & Ribbe, P. H. Quadratic elongation: A quantitative measure of distortion in coordination polyhedral. *Science* **172**, 567-570 (1971).
8. Hoppe, R. Effective coordination numbers (ECoN) and mean active fictive ionic radii (MEFIR). *Z. Kristallogr.* **150**, 23-52 (1979).
